# Enhancing engineering competencies through Outcome-based Education: A mixed-methods study in an intelligent control course

**DOI:** 10.1371/journal.pone.0358558

**Published:** 2026-10-09

**Authors:** Jing Yang, Zhenqing Li, Yoshinori Yamaguchi

**Affiliations:** 1 Faculty of Engineering, Anhui Sanlian University, Hefei, China; 2 School of Optical-Electrical and Computer Engineering, University of Shanghai for Science and Technology, Shanghai, China; 3 Comprehensive Research Organization, Waseda University, Tokyo, Japan; Tel Aviv University, ISRAEL

## Abstract

Bridging the gap between theory and practice remains a persistent challenge in engineering education, especially in complex, algorithm-heavy courses like intelligent control. In such contexts, students often know the abstract concepts but struggle to turn them into workable engineering solutions. Outcome-Based Education (OBE), which focuses on what students can do, offers a promising way forward. However, detailed evidence on how well OBE works at the individual course level in technically demanding fields is still limited. In this quasi-experimental, mixed-methods study, we redesigned an undergraduate intelligent control course around OBE principles, using constructive alignment together with project-based and case-based learning. We compared an intervention cohort (n = 97) with a historical control cohort (n = 89) who had taken the same course the previous year under traditional lectures. Quantitative results from pre- and post-course surveys, exams, and project scores showed meaningful gains for the intervention group across several areas: learning engagement (t = 9.87, p < .001, d = 1.28), practical application skills (t = 13.21, p < .001, d = 1.73), innovation abilities (t = 12.88, p < .001, d = 1.68), final exam scores (84.2 vs. 76.5, p < .001, d = 0.89), and perceived career relevance (t = 11.34, p < .001, d = 1.62). Student feedback further suggested that hands-on projects and regular feedback helped make abstract theories feel concrete and connected to real engineering work. Because we used a historical control group and changed several instructional elements simultaneously, these findings point to a strong positive association rather than definitive cause and effect. Even so, they suggest that a thoughtfully implemented, constructively aligned OBE reform can help build key competencies and bridge theory and practice in advanced science, technology, engineering, and mathematics courses.

## I. Introduction

The rapid evolution of intelligent technologies, such as adaptive control, neural networks, and data-driven optimization, has fundamentally transformed industrial practices and, consequently, the competency profile required of engineering graduates [1]. Modern engineering work increasingly demands the ability to synthesize knowledge across disciplines, tackle complex, ill-defined problems under uncertainty, and innovate through the design and implementation of integrated systems. This shift places immense pressure on engineering education systems to move beyond the transmission of foundational theories and prioritize the development of demonstrable, higher-order competencies [2].

This need to bridge the enduring theory-practice gap is especially acute in advanced, interdisciplinary engineering courses such as intelligent control. This field integrates control theory, artificial intelligence, and systems engineering, requiring students to master abstract mathematical models (e.g., fuzzy logic and neural network architectures) while also developing the practical skills to implement and tune these algorithms for real-world applications [3]. The inherent complexity and cognitive load create a substantial pedagogical challenge. Traditionally, instructor-centered approaches that prioritize exhaustive content coverage and algorithmic derivation tend to dominate such courses [4]. While these methods may support short-term recall for examinations, they often provide limited opportunities for students to engage in the iterative, reflective, and authentic processes of engineering problem-solving, system design, and applied decision-making [5]. Consequently, a disconnect can persist: students may understand the “what” of intelligent control but still lack confidence and proficiency in the “how,” leaving them underprepared for professional practice [6].

In response to these challenges, educational frameworks that emphasize active learning and competency development have gained traction. Among these, Outcome-Based Education (OBE) has emerged as a prominent paradigm, especially in professionally accredited programs [7]. OBE marks a fundamental shift from content-driven to learner-centered instruction. It begins with the clear articulation of what students are expected to know and be able to do upon completion of a learning experience—the intended learning outcomes (ILOs) [8]. The cornerstone of OBE is Biggs’ principle of constructive alignment, which requires systematic coherence among three core elements: the ILOs, the teaching and learning activities (TLAs) designed to help students achieve them, and the assessment tasks (ATs) used to evaluate their level of attainment [9]. When implemented effectively, constructive alignment transforms assessment from a mere grading exercise into an integral part of the learning process, offering continuous feedback and ensuring that all instructional efforts work toward the development of specified competencies [10,11].

Within engineering education, OBE’s alignment with accreditation criteria, such as those from the Accreditation Board for Engineering and Technology that emphasize demonstrable graduate attributes, has spurred widespread program-level adoption [1]. Empirical studies at that level suggest that OBE, especially when combined with active learning approaches like project-based learning, can enhance student engagement and performance [2]. However, translating these principles into course-level practice in technically demanding subjects remains a challenge. For one thing, detailed empirical studies that document and evaluate the systematic operationalization of OBE at the individual course level in complex engineering domains are still limited [12]. For another, many active learning implementations in courses such as intelligent control remain fragmented, rather than being fully embedded within a coherent, outcome-aligned instructional design [13]. Moreover, rigorous mixed-methods evaluations that integrate measurable outcomes with students’ own accounts of the learning process are relatively scarce [14]. This lack of transparent, integrated evidence limits the transferability of course-level OBE designs for high-complexity engineering subjects.

To address these gaps, we implemented and evaluated a course-level, multi-component OBE-oriented redesign in an undergraduate intelligent control course. We used a quasi-experimental, mixed-methods design to generate triangulated evidence, while acknowledging the inferential limitations of using a historical control group. The primary aim was to examine whether this constructively aligned instructional package was associated with improvements in specific engineering competencies and student perceptions of learning.

1. To quantitatively compare the effects of the OBE reform and traditional instruction on students’ self-reported learning engagement, practical application skills, and innovation abilities.
2. To assess differences in objective academic performance (examination and project scores) between an OBE intervention cohort and a historical control cohort.
3. To qualitatively explore students’ learning experiences and perceptions of the OBE approach, thereby elucidating the mechanisms behind any quantitative effects and particularly assessing perceived relevance to career development.

By integrating quantitative measures of competency gain with qualitative insights into the learning process, this study seeks to provide a more holistic and transferable model for strengthening theory-practice integration in complex, practice-oriented engineering courses.

## II. Theoretical framework

This study is grounded in prior literature on OBE and constructive alignment. OBE emphasizes the explicit specification of ILOs and the organization of curriculum, instruction, and assessment around demonstrable student achievement [7,8]. A key theoretical basis for implementing OBE at the course level is Biggs’ principle of constructive alignment, which highlights the need for coherence among ILOs, TLAs, and ATs [9,15]. In engineering education, these principles are often combined with active and practice-oriented pedagogies to strengthen theory–practice integration in technically demanding subjects [12,16]. As illustrated in Fig 1, we operationalized these ideas in the intelligent control course through a framework consisting of four interdependent components: ILOs, modularized course content, teaching and learning strategies, and assessment methods. The framework was designed to ensure that outcomes, learning activities, content organization, and assessment practices worked together to support the development of defined engineering competencies. Conceptually, the framework treats the ILOs as the organizing anchor of the redesign: modularization structures progression toward those outcomes, project- and case-based learning provide authentic contexts in which students can enact the targeted competencies, and aligned formative and summative assessments generate evidence of attainment and feedback for further learning [17]. Rather than viewing these elements as independent techniques, the framework assumes that their educational value arises from their alignment and mutually reinforcing roles within the course design.

**Fig 1:**
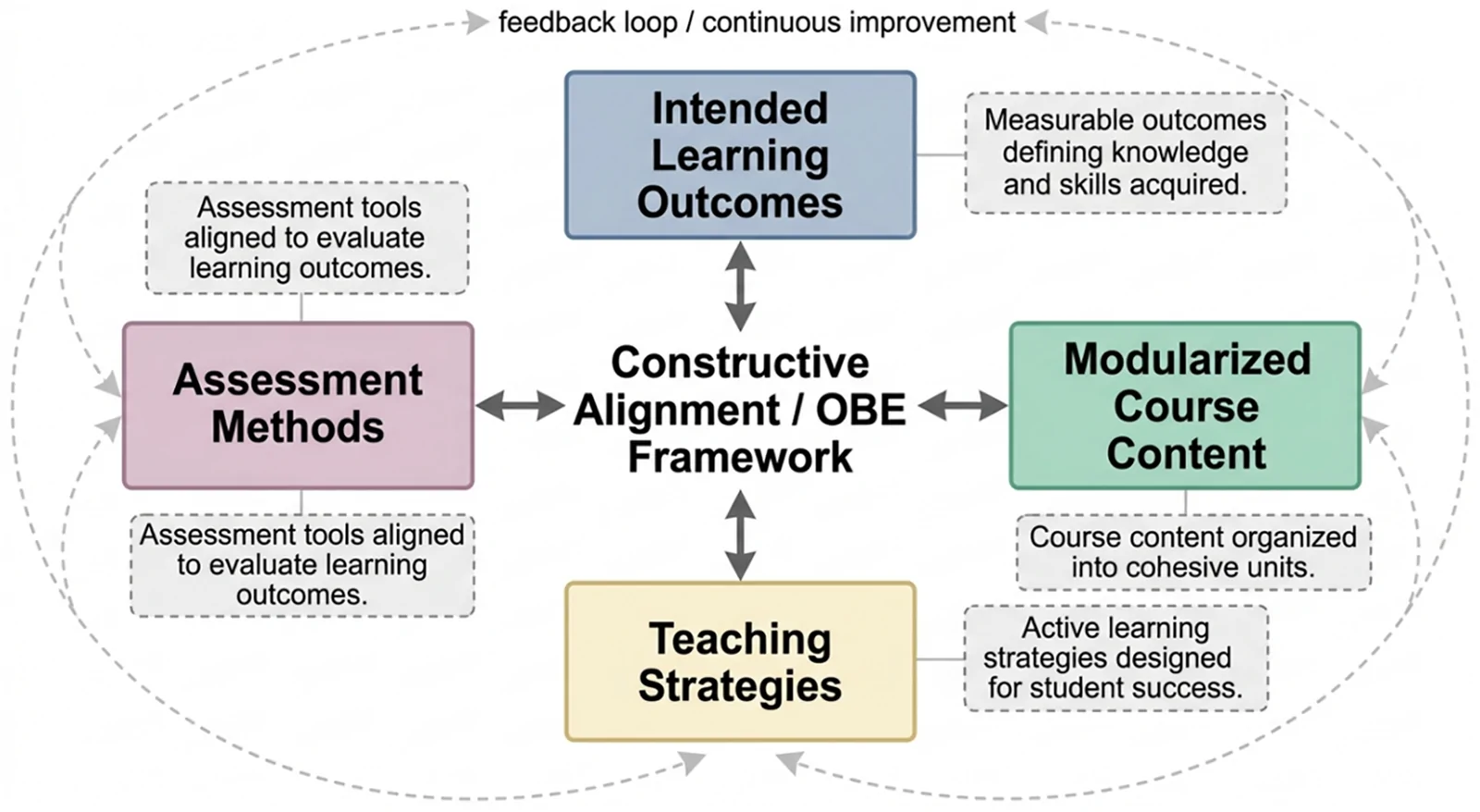
The constructively aligned OBE framework for the intelligent control course.

First, the framework begins by defining ILOs aligned with professional engineering competencies, with emphasis on demonstrable skills such as system design and problem-solving. These outcomes then guide the design of modularized course content, organized into sequential units of progressive complexity. Teaching and learning strategies, including project-based and case-based learning, are used to help students engage with the intended outcomes in applied contexts. Finally, diversified assessment methods, both formative and summative, are used to evaluate integrated competency development. The dashed arrows in Fig 1 indicate feedback loops, through which assessment results inform the refinement of teaching strategies and course design, thereby supporting continuous instructional improvement.

## III. Methods

### A. Study design and setting

We used a quasi-experimental, mixed-methods design to evaluate the effectiveness of an OBE-oriented instructional reform. The quantitative component involved a non-equivalent two-group comparison between an intervention cohort and a historical control cohort, while the qualitative component explored students’ experiences of the redesigned course [14]. Because this comparison did not involve random assignment or a concurrent control group, the design was intended to support cautious comparative interpretation rather than strong causal inference. The study took place in a compulsory undergraduate Intelligent Control course at an engineering-focused university during the 2023–2024 academic year.

### B. Participants and sampling

The target population was third-year undergraduate students majoring in automation and control theory. A total of 97 students enrolled in the course formed the intervention cohort. The historical control cohort consisted of 89 students who had taken the same course in the previous academic year (2022–2023) with the same instructor, where instruction was primarily lecture-based. Although fully matched demographic data were not available for the archival cohort, both groups came from the same degree program, same year level, and same prerequisite structure. This provides partial contextual comparability but does not eliminate the possibility of cohort effects.

All participants in the intervention cohort had completed prerequisite courses in linear control systems and programming. Their mean age was 20.8 years (standard deviation [SD] = 0.7), with 71 male (73.2%) and 26 female (26.8%) students. Institutional archival records for the historical cohort preserved enrollment and performance data but did not retain a fully comparable demographic profile. Participation in the research surveys was voluntary and did not affect course grades. Written informed consent was obtained from all participants in the intervention cohort prior to data collection.

### C. Ethical considerations

This study was approved as an educational research project by *** University (Project Approval No.: [Blinded for Review]). Because the study involved only anonymous surveys and anonymized archival grades, and posed minimal risk, formal institutional review board approval was not required. Written informed consent was obtained from all intervention cohort participants. For the historical cohort, consent was waived due to the use of anonymized archival data. All procedures followed the Declaration of Helsinki. Confidentiality was protected through data anonymization, and course grades were not affected by survey participation.

### D. Outcome-based education intervention

The instructional intervention was systematically designed according to the principle of constructive alignment [9]. The redesigned course was implemented by the same instructor who had taught the historical control cohort, which helped maintain continuity in disciplinary background and course expectations across the two offerings. Before the intervention semester, the instructor engaged in structured preparation for the OBE-oriented redesign, including aligning course-level intended learning outcomes with module content, project tasks, and assessment criteria. Although the instructor did not participate in a formal standalone OBE certification program, the redesign was informed by prior teaching experience, institutional curriculum-development requirements, and deliberate pre-course planning focused on outcome alignment and competency-oriented instruction. Importantly, the reform should be understood as a bundled instructional package rather than a set of isolated techniques, because outcome specification, modularized content, project-based learning, case-based learning, teamwork, and diversified assessment were intentionally implemented together. The instructor has a disciplinary background in automation and control engineering and has extensive experience teaching undergraduate control-related courses. The instructor had taught the Intelligent Control course for 9 years and had also participated in curriculum development and course-design activities.

1. Defining ILOs: Four course-level competencies were established: (1) Mastery of intelligent control theory, (2) Practical application and system design ability, (3) Innovation and problem-solving competence, and (4) Teamwork and communication skills.
2. Modularizing Course Content: The syllabus was reorganized into four aligned modules: Foundational Theory, Intelligent Control Algorithms, Engineering Applications, and Emerging Technologies.
3. Designing TLAs: Traditional lectures were supplemented with case-based analyses and a core semester-long, team-based design project. Students worked in groups of 4–5 to design, simulate (using MATLAB/Python), and optimize intelligent controllers for realistic engineering problems.
4. Aligning ATs: A diversified assessment scheme was implemented, including formative quizzes, a final examination, and comprehensive project evaluations assessing system design, algorithm implementation, and report quality.

### E. Data collection

Data were collected from multiple sources to ensure methodological triangulation.

1. Structured Questionnaire: A self-administered survey was distributed to the intervention cohort at the beginning (pre-test) and end (post-test) of the semester. It contained 52 items across five constructs measured on a 5-point Likert scale (1 = Strongly Disagree, 5 = Strongly Agree): Learning Engagement (12 items, α = 0.89); Theoretical Understanding (10 items, α = 0.86); Practical Application Skills (12 items, α = 0.91); Innovation & Problem-Solving (10 items, α = 0.88); and Career Relevance Perception (8 items, α = 0.84). To improve transparency, representative items included statements such as “I actively participated in discussions and project activities” (Learning Engagement), “I can explain the principles behind the major intelligent control methods taught in this course” (Theoretical Understanding), “I can design and implement an intelligent controller for a practical task” (Practical Application), “I can compare alternative control strategies for an open-ended engineering problem” (Innovation & Problem-Solving), and “This course helped me understand the relevance of intelligent control to my future career” (Career Relevance). To strengthen the validity of the instrument, several aspects of validity were considered. Face validity was addressed through expert review of item clarity, wording, and appropriateness for the intelligent control course context. Content validity was examined by two faculty members with relevant expertise in engineering education and course design, who reviewed whether the questionnaire items adequately reflected the targeted learning dimensions. Construct validity was further examined through Exploratory Factor Analysis (EFA), as reported in the Results section. Of the 52 questionnaire items, 22 competency-focused items corresponding to the four intended learning outcome dimensions were included in the EFA; items measuring learning engagement and career relevance were treated as separate perceptual constructs and were not included in this factor analysis. All questionnaire items were positively worded. This feature is acknowledged as a potential source of acquiescence bias and is explicitly addressed in the Limitations and Future Work section.
2. Academic Performance Records: Final examination scores and detailed project evaluation scores were collected for both the intervention and historical control cohorts. The same instructor taught both course offerings, and the examinations were aligned with the same core course objectives. However, because the assessments were administered in different academic years, perfect equivalence of testing conditions cannot be assumed, and the results should be interpreted with this limitation in mind.
3. Qualitative Feedback: Open-ended questions at the end of the post-test survey solicited student reflections on their learning experiences, perceived challenges, and views of the OBE-oriented redesign. The qualitative component was used to enrich the interpretation of the quantitative findings by examining how students described the value of projects, feedback, collaboration, and the integration of theory and practice.

### F. Data analysis

Quantitative data were analyzed using Statistical Package for the Social Sciences (SPSS) 27.0 and R 4.2.1. Descriptive statistics summarized all variables. Paired-sample t-tests assessed within-cohort changes (pre-test vs. post-test) for the intervention group. Independent-sample t-tests compared final examination scores between the intervention and historical control cohorts, but these between-cohort comparisons were interpreted cautiously given the historical-control design. Multiple linear regression was used to identify predictors of perceived career relevance. EFA with Promax rotation was conducted on the 22 competency-focused questionnaire items to examine the construct structure of the ILOs. Effect sizes were calculated using Cohen’s d, and statistical significance was set at p < .05. Paired-sample t-tests were selected because the pre-test and post-test scores were obtained from the same students, whereas independent-sample t-tests were used because the intervention and historical cohorts represented two separate groups. The use of these inferential tests was supported by the comparison structure of the data and by preliminary examination of distributional characteristics, including skewness and kurtosis values, which did not indicate severe departures from normality. Because the historical control cohort did not complete the same baseline questionnaire, comparable baseline covariates were unavailable for a baseline-adjusted ANCOVA/MANCOVA analysis. In addition, the primary between-cohort comparison involved a single common outcome, final examination score. Therefore, an independent-samples t-test was selected as the more appropriate analysis for this comparison. For the five paired pre-post comparisons in the intervention cohort, the family-wise error rate was addressed using the sequential Holm-Bonferroni procedure; all five comparisons remained statistically significant at the .05 level after correction. Assumptions were evaluated in relation to each analysis: approximate normality was assessed from distributional characteristics and residual diagnostics, homogeneity of variance was assessed for the between-group comparison, and the regression analysis was evaluated for linearity, independence of errors, homoscedasticity, approximate normality of residuals, and multicollinearity [18].

Qualitative data from open-ended responses were analyzed using thematic analysis following the six-phase approach by Braun and Clarke [19]. Two researchers independently coded a subset of responses to enhance reliability (intercoder agreement κ = 0.86). Themes were then refined through discussion and used to contextualize the quantitative results. Because the qualitative strand was intended to complement rather than independently generalize the findings, the resulting themes were interpreted as explanatory patterns within this course context.

## IV. Results

A total of 97 students in the intervention cohort completed both the pre- and post-course surveys, as well as all course assessments. All data from the historical control cohort (n = 89) were available for analysis.

### A. Descriptive statistics and preliminary analyses

Table 1 presents the descriptive statistics for all self-reported measures from the intervention cohort at pre-test and post-test. Post-test mean scores showed substantial increases across all five competency and perception dimensions compared to baseline scores.

**Table 1:** Descriptive statistics for pre-test and post-test measures in the intervention cohort (N = 97).

Measure	Pre-test *M (SD)*	Post-test *M (SD)*	Possible Range	Skewness	Kurtosis
Learning Engagement	3.21 (0.74)	4.12 (0.68)	1–5	–0.32	0.41
Theoretical Understanding	3.45 (0.71)	4.05 (0.62)	1–5	–0.41	0.38
Practical Application	2.98 (0.80)	4.18 (0.59)	1–5	–0.28	0.52
Innovation & Problem-Solving	3.02 (0.77)	4.22 (0.56)	1–5	–0.35	0.47
Career Relevance	3.10 (0.82)	4.25 (0.61)	1–5	–0.39	0.44

*Note:* All measures used a 5-point Likert scale (1 = strongly disagree, 5 = strongly agree). M = Mean; SD = standard deviation.

### B. Changes in self-reported competencies and engagement

To evaluate the impact of the OBE intervention, paired-sample t-tests were conducted on the pre- and post-test scores of the intervention cohort. As shown in Table 2, statistically significant improvements with large effect sizes (Cohen’s d > 0.80) were observed in all four competency domains and career relevance. After application of the Holm-Bonferroni correction across the five paired comparisons, all five effects remained statistically significant at the .05 family-wise level.

**Table 2:** Paired-sample t-test results for learning and perceptual outcomes.

Dimension	*t*	*df*	*p*	Cohen’s *d*	95% CI for *d*
Learning Engagement	9.87	96	< .001	1.28	[0.95, 1.61]
Theoretical Understanding	7.45	96	< .001	0.94	[0.67, 1.21]
Practical Application	13.21	96	< .001	1.73	[1.42, 2.04]
Innovation & Problem-Solving	12.88	96	< .001	1.68	[1.37, 1.99]
Career Relevance	11.34	96	< .001	1.62	[1.32, 1.92]

Note: All p-values are two-tailed. CI = confidence interval.

### C. Comparative academic performance: Intervention vs. historical control

An independent-samples t-test was conducted to compare the final examination performance of the intervention cohort with that of the historical control cohort. The intervention cohort (M = 84.2, SD = 8.1) scored significantly higher than the control cohort (M = 76.5, SD = 9.4), t(184) = 6.12, p < .001, Cohen’s d = 0.89, 95% CI [0.60, 1.18]. This performance difference was more pronounced for application-oriented questions (mean difference = 8.7 points) than for theoretical questions (mean difference = 4.2 points). However, because the comparison involved a historical rather than a concurrent control group, this result should be interpreted as a meaningful between-cohort difference associated with the reform, rather than as definitive proof that the redesign alone caused the score increase.

The project assessment results for the intervention cohort (evaluated across 24 groups) are presented in Table 3. High levels of achievement were observed across all evaluation dimensions. Inter-rater reliability for the project scoring was excellent (intraclass correlation coefficient [ICC] = .91, 95% CI [.85, .95]).

**Table 3:** Project assessment results by dimension (N = 24 groups).

Dimension	*M* (out of 25)	*SD*	Min	Max	% ≥ Target
System Design	20.4	3.2	14	25	79.2%
Algorithm Implementation	22.8	2.1	18	25	95.8%
Performance Evaluation	19.7	3.5	12	24	75.0%
Report Quality	21.2	2.8	15	25	83.3%

*Note:* Target score = 20 (80% of maximum). Inter-rater reliability: ICC = .91, 95% CI [.85, .95].

A moderate positive correlation was found between the overall project scores and final examination scores in the intervention cohort, *r* = .52, *p* < .001.

### D. Predictors of perceived career relevance

A multiple linear regression was performed to predict post-test scores on Career Relevance Perception. The model included pre-test relevance scores, overall project performance, and post-test learning engagement scores as predictors. The overall regression was statistically significant, R^2^ = .44, F(3, 93) = 24.17, *p* < .001. As shown in Table 4, both project performance (*β* = .38, *p* < .001) and learning engagement (*β* = .32, *p* = .002) were significant positive predictors.

**Table 4:** Multiple regression analysis predicting career relevance perceptions.

Predictor	*B*	*SE B*	*β*	*t*	*p*	95% CI for *B*
Constant	1.24	0.38		3.26	.002	[0.49, 1.99]
Pre-test Relevance	0.21	0.09	.18	2.33	.022	[0.03, 0.39]
Project Performance	0.38	0.10	.38	3.80	< .001	[0.18, 0.58]
Learning Engagement	0.32	0.10	.32	3.20	.002	[0.12, 0.52]

*Note: R*^2^ = .44, *F*(3, 93) = 24.17, *p* < .001. *B* = unstandardized coefficient; *β* = standardized coefficient.

Regression diagnostics indicated that the model assumptions were adequately satisfied. The Durbin–Watson statistic was 1.93, indicating no substantial autocorrelation. VIF values ranged from 1.12 to 1.46, suggesting no problematic multicollinearity. Residuals were approximately normally distributed, and inspection of residual plots indicated no substantial violations of homoscedasticity or linearity.

### E. Exploratory factor analysis of learning outcomes

An EFA was conducted on the 22 competency-focused questionnaire items to examine the underlying structure of the measured learning outcomes. The Kaiser-Meyer-Olkin measure (.87) and Bartlett’s test of sphericity (*χ²*(231) = 1245.32, *p* < .001) confirmed the data’s suitability. Principal axis factoring with Promax rotation yielded a clear four-factor solution, explaining 68.4% of the total variance. As presented in Table 5, all items loaded strongly (> .40) on their hypothesized factors, confirming the construct validity of the four intended learning outcome dimensions: Theoretical Understanding, Practical Application, Innovation & Problem-Solving, and Teamwork & Communication.

**Table 5:** Factor loadings from exploratory factor analysis (Promax rotation).

Item	Factor 1	Factor 2	Factor 3	Factor 4
**Theoretical Understanding**				
TU1: Explain fuzzy logic principles	.78	.12	.05	–.03
TU2: Describe neural network structures	.82	.08	.10	.04
**Practical Application**				
PA1: Implement PID controller	.09	.85	.11	.07
PA2: Tune control parameters	.14	.79	.16	.09
**Innovation & Problem-Solving**				
IPS1: Propose alternative solutions	.11	.18	.82	.12
IPS2: Optimize system performance	.08	.22	.76	.15
**Teamwork & Communication**				
TC1: Collaborate effectively	.05	.09	.14	.88
TC2: Present technical content	.07	.11	.08	.83

*Note:* Factor loadings > .40 are in bold. Cross-loadings were all < .30.

### F. Thematic analysis of qualitative feedback

Thematic analysis of 87 open-ended responses from the intervention cohort identified four primary themes regarding the learning experience. Overall, students described the redesigned course as more demanding than conventional lecture-based instruction but also more relevant to authentic engineering practice. These thematic findings help explain why the largest gains were observed in practical application, innovation, and career relevance. As a worked example of the analytic process, the response “Before the project, fuzzy logic was just a set of mathematical formulas to me” was initially coded as “abstract theory,” while the continuation “implementing a fuzzy controller... made me understand why membership functions work” was coded as “understanding through implementation.” These initial codes were then grouped under the broader category of theory–practice integration and ultimately contributed to the theme “Project-Driven Conceptual Understanding.” This example illustrates how raw student responses were progressively interpreted from initial coding to thematic categorization.

1. Project-Driven Conceptual Understanding: Students frequently reported that implementing algorithms within practical projects made abstract theories more concrete and meaningful. The recurring pattern in these responses suggested that mathematical concepts became easier to understand once students had to test, debug, and refine controller behavior in realistic tasks. *Student quote: “Before the project, fuzzy logic was just a set of mathematical formulas to me. Actually, implementing a fuzzy controller for a temperature regulation problem made me understand why membership functions work the way they do.”* (Student 24)
2. Formative Feedback as a Learning Catalyst: Students highlighted iterative feedback during project milestones as an important mechanism for learning. Rather than merely identifying mistakes, feedback was described as helping students understand underlying engineering reasoning, justify design choices, and improve performance across successive project stages. *Student quote: “The teaching assistant feedback on our first milestone didn’t just say ‘wrong tuning’; it showed us how the gain values affected system stability. That made me rethink my whole approach.”* (Student 89)
3. Benefits of Collaborative Learning: Group work was described as instrumental in developing both technical integration skills and communication abilities. Students indicated that teamwork enabled them to distribute programming, modelling, and analytical tasks while also learning how to explain technical decisions to peers. *Student quote: “My partner was good at Python, and I understood the control theory better. Together we built a better system than either of us could alone.”* (Student 12)
4. Challenge-to-Growth Trajectory: Many responses reflected an initial sense of difficulty when students encountered open-ended design tasks, followed by a marked increase in confidence by the course’s end. This pattern suggests that the perceived challenge of the redesigned course may have functioned as a productive source of growth rather than merely an obstacle. *Student quote: “In the first two weeks, I felt lost. The project seemed too big. But by week 8, when our simulation finally ran correctly, I realized how much I had actually learned.”* (Student 33)

### V. Discussion

#### A. Key findings

This mixed-methods study provides evidence that a systematically implemented, constructively aligned OBE-oriented reform was associated with enhanced engineering competencies and more positive learning perceptions in a complex undergraduate intelligent control course. Quantitative results showed substantial improvements in students’ self-reported learning engagement, theoretical understanding, and especially practical application and innovation skills. These subjective gains were accompanied by stronger final examination performance than that observed in a historical control cohort. Qualitative responses further suggested that projects, collaboration, and iterative feedback helped students connect abstract theory with engineering practice. These results are broadly consistent with the concerns outlined in the Introduction regarding the theory–practice gap in engineering education and with previous studies suggesting that active and outcome-oriented approaches can support stronger student engagement and performance. The improvement in engagement and applied performance is consistent with prior engineering-education research emphasizing active student participation and authentic problem solving as mechanisms for strengthening learning and performance [1]. The qualitative emphasis on projects, collaboration, and iterative feedback also accords with problem- and project-based learning literature that highlights the role of collaborative, practice-oriented tasks in integrating disciplinary knowledge with engineering action [13,17].

The most pronounced improvement was observed in practical application skills (Cohen’s d = 1.73), which directly addresses a longstanding criticism of engineering education: the difficulty of connecting formal theory to authentic practice. At the same time, the magnitude of some effect sizes should be interpreted cautiously, given the close alignment between the intervention and the measured outcomes, the relatively modest pre-test baselines for some dimensions, and the use of self-reported measures. The overall pattern of findings also supports the relevance of constructive alignment in technically demanding engineering courses. This finding is also compatible with constructive-alignment theory, which proposes that learning outcomes, teaching activities, and assessment should be deliberately coordinated so that students repeatedly practice and demonstrate the competencies the course intends to develop [15].

### B. Implications

Practical implications of this work are relevant for engineering educators. First, the study offers a course-level example of how ILOs, modularized content, active learning activities, and assessment can be deliberately aligned around demonstrable competencies. Second, the findings highlight the likely value of iterative project work and feedback in supporting theory-practice integration in advanced engineering subjects. Third, the collaborative dimension of the redesign suggests that team-based tasks can simultaneously support technical learning and communication development. However, these implications should be understood as design-informed lessons from a bundled reform package rather than as evidence that any single component alone is sufficient. These implications parallel prior work showing that coherent project/problem-based designs can support complex engineering learning when problem design, collaboration, and assessment are intentionally structured rather than added as isolated activities [16].

### C. Limitations and future work

This study has several limitations. First, the use of a historical control group, while pragmatically necessary, introduces potential confounding from cohort differences, year-specific contextual factors, and possible differences in assessment conditions. Second, several important outcomes were measured through self-report scales, which may capture students’ perceptions of growth more directly than objectively verified competency gain. Third, because the intervention combined outcome specification, modularized content, project-based learning, case-based learning, collaborative work, and diversified assessment, it is not possible to isolate the independent contribution of each component. Fourth, full demographic equivalence testing between cohorts was not possible because archival records for the historical group were limited. Finally, the study was conducted in a single institution and one course, which constrains generalizability. In addition, all questionnaire items were positively worded, which may have increased susceptibility to acquiescence bias and could have inflated favourable self-reports. Future questionnaire designs should consider a carefully balanced use of positively and negatively worded items, together with appropriate validation, to reduce this potential response tendency.

Future research should build on these findings through more rigorous and more transparent comparative designs. Concurrent comparison groups, multi-course replication, richer objective performance indicators, and longitudinal follow-up into capstone projects or early professional contexts would strengthen the evidence base. Future studies could also examine which specific elements within a bundled OBE-oriented redesign—such as project work, formative feedback, or assessment alignment—contribute most strongly to different kinds of student outcomes.

## VI. Conclusion

In conclusion, this study suggests that OBE, implemented through deliberate constructive alignment at the course level, may be a useful strategy for strengthening engineering competency development in a complex, application-oriented domain. It was associated with improved practical skills, stronger perceived integration of theory and practice, greater innovation-related confidence, and more positive perceptions of career relevance. At the same time, the historical-control design and bundled nature of the intervention mean that these findings should be interpreted cautiously. For educators seeking to narrow the theory-practice gap in advanced science, technology, engineering, and mathematics courses, this study offers a promising and transparent course-level model that merits further testing and refinement.
